# Nanomechanical Characterization of E-Cigarette-Induced Lung Endothelial Dysfunction: Roles of Cortactin and Mitochondrial Reactive Oxygen Species

**DOI:** 10.3390/ijms262412104

**Published:** 2025-12-16

**Authors:** Mounica Bandela, Xue Geng, Joe G. N. Garcia, James C. Lee, Steven M. Dudek

**Affiliations:** 1 Department of Biomedical Engineering, College of Engineering, University of Illinois Chicago, Chicago, IL 60607, USA; 2 Division of Pulmonary, Critical Care, Sleep and Allergy, Department of Medicine, University of Illinois Chicago, Chicago, IL 60612, USA; sdudek@uic.edu; 3 Department of Bioengineering and Biomedical Sciences, Lawrence Berkeley National Laboratory, Berkeley, CA 94720, USA; 4 Department of Molecular Medicine, Scripps Research Institute, University of Florida, Jupiter, FL 33458, USA

**Keywords:** atomic force microscopy, elastic properties, mitochondrial dysfunction, permeability

## Abstract

E-cigarettes (E-cigs) are increasing in popularity and are considered a potentially safer alternative to traditional cigarettes. However, prior studies have demonstrated that inhalation of nicotine-containing e-cigs can cause substantial pathophysiologic changes, and “vaping” of some substances has led to severe lung damage. Our group recently described the role of cortactin (*CTTN*), a cytoskeletal actin-binding regulatory protein, in mediating cigarette smoke (CS) and E-cig-induced lung endothelial apoptosis and mitochondrial dysfunction. In the current study, we advance this work by characterizing the effects of E-cig on lung endothelial nanomechanical properties and barrier function. Lung EC exposure to E-cig extract (50 µg/mL) resulted in disruption of endothelial barrier properties as assessed by Electric Cell–Substrate Impedance Sensing (ECIS). Since excess mitochondrial reactive oxygen species (mitoROS) is an important marker of mitochondrial dysfunction, we next assessed the effect of Mito-TEMPO (10 µM, 3 h), a cell-permeable antioxidant, on E-cig-induced endothelial permeability. Pretreatment with Mito-TEMPO provided EC barrier protection after E-cig challenge, suggesting a key role of mitoROS in E-cig-induced EC permeability. E-cig exposure induces cytoskeleton rearrangement, leading to gap formation in lung EC, and significantly alters EC elastic properties as assessed by atomic force microscopy (AFM). Reduction in *CTTN* expression by siRNA further augmented the injurious effects of E-cig on EC permeability and elastic properties. This is the first study to explore the role of CTTN in evaluating the effect of E-cigarette exposure on the lung endothelium using AFM and provides novel mitochondrial and biophysical characterization of the effects of E-cig exposure on human lung EC. This work advances our understanding of the pathophysiologic effects of E-cig exposure.

## 1. Introduction

Electronic cigarettes (E-cigs) are gaining popularity, especially among youth, and have been considered as a potentially safer alternative to traditional cigarettes [1]. E-cigarettes (E-cigs) first entered the US market in 2006, while pod mode forms such as “JUUL” were introduced in 2015 [2]. In 2019 the Centers for Disease Control and Prevention (CDC) [3] and Food and Drug Administration (FDA) [4] declared an “E-cigarette epidemic” due to a national outbreak of E-cig-induced acute lung injury (EVALI) cases [5]. E-cig inhalation causes lung tissue damage, thereby increasing the risk of chronic lung disease [6,7,8], including Chronic Obstructive Pulmonary Disorder (COPD). The underlying mechanisms by which E-cigs contribute to lung disease are incompletely understood; however, recent reports suggest that E-cigs cause inflammation, apoptosis, and tissue damage [9,10,11].

Despite the growing interest in their potential negative effects, it is unknown how E-cig exposure may alter the biomechanical properties of lung endothelial cells (ECs), which play a critical role in maintaining vascular barrier integrity [12]. EC dysfunction is a hallmark of various pulmonary diseases, including those associated with cigarette and E-cig exposure [12,13]. Cytoskeletal changes are central to EC responses to injurious stimuli such as cigarette smoke (CS) [14]. CS exposure directly modulates EC barrier permeability, necrosis, and apoptosis through multiple signaling pathways involving RhoA, FAK, and p38, all of which participate in cytoskeletal rearrangements [15,16]. Our group has identified cortactin (CTTN), a central regulator of the actin cytoskeleton, as an important modulator of lung EC function and lung injury responses [17]. Understanding the potential detrimental effects of E-cigs on EC cytoskeletal organization and associated biomechanical properties may provide novel insights into EVALI pathophysiology.

In prior work, we have shown that atomic force microscopy (AFM) is an invaluable tool to characterize nanoscale mechanical changes in living cells, including endothelium [12,13]. AFM offers the sensitivity to detect early or subtle cytoskeletal disruptions that may precede overt morphological damage. AFM is a high-resolution scanning probe technique designed to investigate interatomic forces and surface topography at the nanoscale [13]. Using force–indentation curves and contact mechanics models such as Hertz or Sneddon, AFM enables the precise measurement of local elastic responses from cell membranes and underlying cytoskeletal structures [12,13,18]. For most cell types, Young’s modulus typically ranges between 1 and 100 kPa [19,20], with stiffness largely governed by the integrity of the actin cytoskeleton and internal mechanical forces. These nanomechanical insights are critical, as alterations in cell stiffness are closely linked to changes in cellular function and are often associated with pathological processes. For example, malignant cancer cells may appear morphologically similar to benign ones; they can often be differentiated by their lower elastic modulus—reflecting cytoskeletal remodeling that facilitates invasion and metastasis [21]. This softening is often driven by upregulation of actin-regulating proteins such as Arp2/3, cortactin, and cofilin, promoting the formation of invasive structures like podosomes and invadopodia [21,22,23,24]. These findings highlight AFM’s sensitivity in detecting biomechanical signatures of disease, particularly those driven by cytoskeletal reorganization. However, accurate interpretation of AFM-derived mechanical data depends on the probe geometry, indentation models, applied forces, and cell type, along with calibration for reproducibility.

In this study, we apply AFM to evaluate the nanomechanical properties of lung endothelial cells exposed to E-cigarette extract. We characterize cell stiffness through spatial force mapping and analyze the elastic modulus, providing insight into how E-cigarette exposure disrupts endothelial mechanical integrity. To our knowledge, this work is the first to use atomic force microscopy to characterize the nanomechanical changes induced in the lung endothelium by E-cig exposure, including pathophysiologic alterations in endothelial stiffness and cytoskeletal integrity. Our work reveals novel mechanobiological insights into early injury pathways underlying E-cig-associated lung disease and further advances our understanding of the pathophysiological effects of E-cigs on the lung vasculature.

## 2. Results

### 2.1. E-Cigarette Exposure Induces Cytoskeletal Rearrangement and Gap Formation in Lung ECs

**Figure 1:**
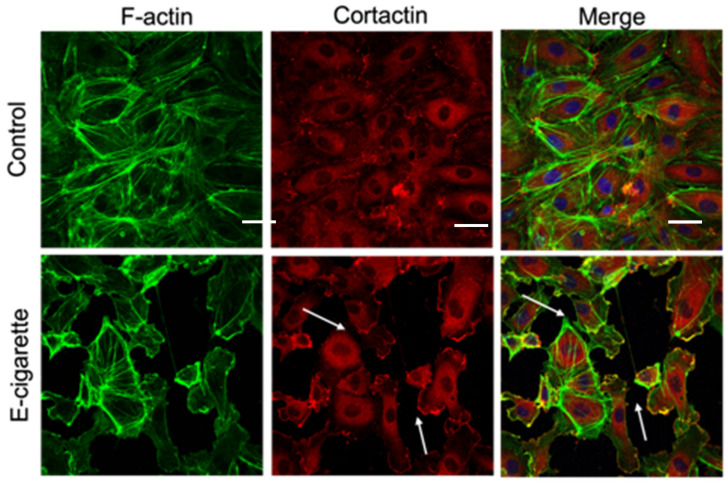
E-cigarette induces loss of cell–cell interaction in endothelial cells. HPAECs were treated with vehicle or E-cig (25 μg/mL) for 3 h, fixed, and subjected to immunofluorescence analysis. Confocal images were taken at 40× after staining with Alexa488-CTTN (red), Alex 594-Phalloidin (F-actin staining, green) and DAPI (nucleus staining, blue). Arrows indicate areas of gap formation and loss of cell–cell interactions. Scale bar is 20 μm.

We first evaluated the effects of E-cigs on the structure of critical cytoskeletal proteins cortactin (CTTN) and actin, in cultured human lung ECs. As revealed by immunofluorescence imaging, E-cig stimulation of HPAEC causes redistribution of actin and CTTN that decreases their expression at the cell periphery (Figure 1). This pattern is similar to that we previously reported in response to CS exposure and is associated with decreased cell–cell interaction, gap formation between cells, and increased EC permeability [14,25].

### 2.2. Trans-Endothelial Resistance Is Decreased by E-Cigarette Exposure in a Dose-Dependent Manner in Lung ECs

**Figure 2:**
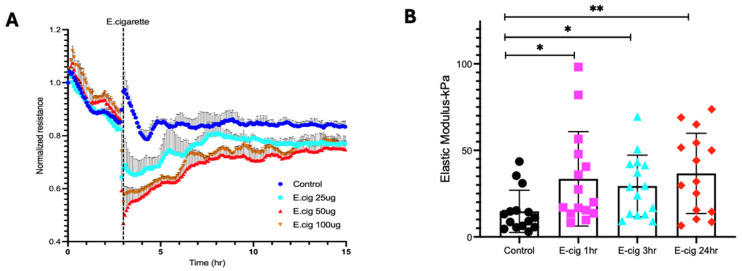
(**A**): E-cigarette causes loss of endothelial barrier function. HPAECs were grown on gold electrodes to ~90% confluence and then challenged with E-cigarette at three different concentrations (25 µg/mL, 50 µg/mL, and 100 µg/mL). HPAEC monolayer permeability dynamics were assessed over 15 h by continuous TEER measurements. A representative ECIS tracing is shown. (**B**): Elastic modulus of cytoplasm of HPAECS in response to E-cig in a time-dependent manner. E-cig induces an increase in Young’s modulus in HPAECs compared to control at 1 h, 3 h, and 24 h. Shown is a representative bar graph for 15–20 cells per condition. * *p* < 0.05; ** *p* < 0.005.

Trans-endothelial electrical resistance (TEER) measurements were obtained to investigate the effects of E-cig on EC barrier function, a primary physiologic role for the endothelium [18]. Dose-dependent E-cig exposure resulted in prolonged disruption of EC barrier function with exposure to 25 µg/mL or higher of E-cig (Figure 2A).

### 2.3. Elastic Modulus Magnitude Is Increased in Lung ECs by E-Cig Exposure

We next characterized the mechanical properties of ECs via AFM. Sub-confluent ECs were first imaged using AFM contact mode, and force curves were subsequently generated. AFM analyses of untreated cells were used as controls and compared to ECs exposed to E-cig in a time-dependent manner (1 h, 3 h, and 24 h). AFM measurements show that E-cig exposure significantly increased the cell stiffness of ECs as compared to controls without E-cig exposure (Figure 2B). For the maximum loads applied during our experiments (~2 nN), the indentation made on ECs by the AFM tip was typically ~200–600 nm.

### 2.4. Cortactin Expression Modulates the Lung Barrier Effects of E-Cigarettes

**Figure 3:**
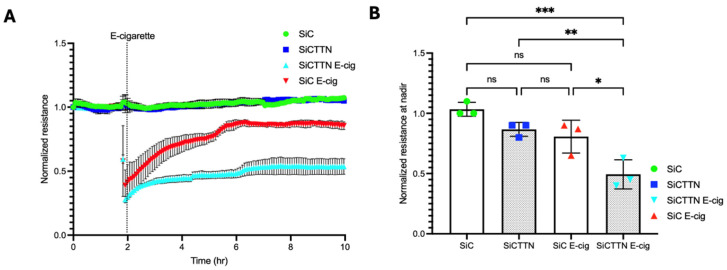
E-cig-induced permeability is regulated by CTTN expression in HPAEC. (**A**): HPAECs grown on ECIS plates were transfected with control or CTTN-specific siRNA and then challenged with E-cig (25 µg/mL). TEER was measured, and the resistance in each well was normalized. (**B**): Quantification of normalized resistance at nadir. Shown is a tracing from a representative experiment. * *p* < 0.05; ** *p* < 0.005; *** *p* < 0.0005, and ns-nonsignificant.

Lung EC permeability is regulated by CTTN expression and function [14,26,27]. To investigate its role in lung EC barrier integrity during E-cig exposure, ECs were transfected with control or *CTTN* siRNA for 48 h and then seeded on TEER plates. Western blotting confirmed a significant reduction in protein expression of CTTN via siRNA [14], as shown in our previous work. TEER measurements demonstrated that E-cig-induced barrier disruption is significantly increased in ECs treated with CTTN siRNA (Figure 3). These data support an important role for CTTN expression in attenuating EC barrier disruption caused by E-cigs.

### 2.5. Role of MitoROS in E-Cig-Induced Lung EC Permeability

**Figure 4:**
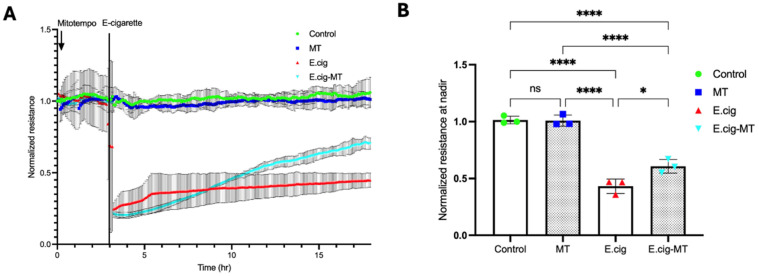
MitoTEMPO restores lung endothelial barrier function after E-cig challenge. (**A**): HPAECs grown on gold electrodes to ~90% confluence were pretreated with MitoTEMPO (20 µM for 3 h), followed by 25 µg/mL of E-cig. Real-time changes in TEER were measured continuously over 15 h using ECIS. (**B**): A statistical analysis was performed at the TEER nadir; normalized resistance data were obtained from three independent experiments. * *p* < 0.05; **** *p* < 0.0001, and ns-nonsignificant.

Our recent report demonstrated a novel role for CTTN in mitoROS generation and lung endothelial apoptosis [14]. MitoTEMPO is a scavenger of mitoROS and an effective tool for assessing its functional significance [28]. Here, we investigated the role of mitoROS in E-cig-induced lung EC permeability by pretreating ECs with MitoTEMPO (20 µM for 3 h) prior to E-cig challenge (25 µg/mL). TEER measurements demonstrated partial attenuation of E-cig-induced barrier disruption in ECs pretreated with MitoTEMPO, confirming an important functional role for mitoROS in E-cig-induced barrier disruption (Figure 4).

### 2.6. Mitochondrial ROS Participates in E-Cig-Induced Elastic Modulus Changes in Lung ECs

**Figure 5:**
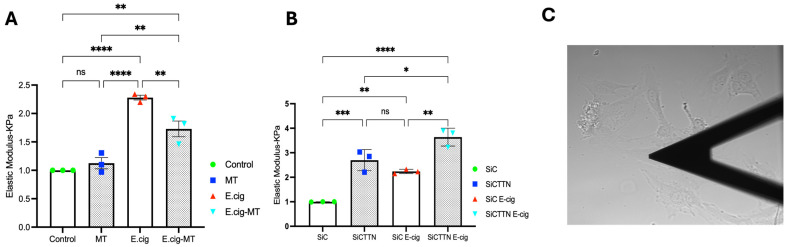
(**A**): MitoTEMPO attenuates EC elastic modulus responses to E-cig. E-cig induces a significant increase in Young’s modulus in HPAECs compared to control, but pretreatment with MitoTEMPO (20 µM, 3 h) lowers the elastic modulus in E-cig-induced ECs. Shown is a representative bar graph from three independent experiments (15–20 cells per condition/experiment). (**B**): Elastic modulus responses to E-cig are enhanced by cortactin depletion in lung endothelial cells. Inhibition of CTTN expression by siRNA (48 h) enhances this elastic modulus response to E-cig in HPAECs compared to control. The bar graph represents data from three independent experiments (15–20 cells per condition/experiment). (**C**): White field image showing a cantilever tip measuring the elastic modulus of HPAECs. * *p* < 0.05; ** *p* < 0.005; *** *p* < 0.0005; **** *p* < 0.0001, and ns-nonsignificant.

We next characterized the role of mitochondrial ROS in EC nanomechanical responses to E-cig using AFM. Sub-confluent ECs were first imaged using contact mode and then force–distance maps were subsequently generated. AFM analyses of untreated cells were used as controls and compared to ECs pretreated with MitoTEMPO (20 µm, 3 h) and then challenged with E-cig for 1 h. AFM measurements were used to generate elastic maps to determine Young’s modulus values. For maximum loads applied during our experiments (~2 nN), the deformation of the ECs by the AFM tip was typically ~200–600 nm. Indentation depths (200–600 nm) correspond to <10% of the endothelial cell height (6–8 μm). No plastic deformation was observed during unloading. This approach ensured that the Hertz model remained valid within our indentation range. The EC elastic modulus was significantly increased by E-cig exposure, but this increase was significantly attenuated in ECs pretreated with MitoTEMPO, supporting an important functional role for mitochondrial ROS in E-cig-induced EC nanomechanical responses (Figure 5A).

### 2.7. CTTN Expression Regulates E-Cig-Induced Elastic Modulus Responses in Lung Endothelial Cells

We further explored the role of CTTN in E-cig-induced elastic modulus responses using AFM. ECs were transfected with control or *CTTN*-specific siRNA for 48 h and then challenged with E-cig for 1 h. Young’s modulus was then determined as above. The elastic modulus was again increased upon E-cig challenge. This increase was significantly enhanced in siCTTN cells, further demonstrating an important role for CTTN in regulating E-cig-induced EC nanomechanical responses (Figure 5B).

## 3. Discussion

### 3.1. Background

In 2019 the CDC/FDA declared an E-cigarette epidemic due to a national outbreak of E-cigarette-induced acute lung injury cases [E-cig or Vaping product use-associated acute lung injury (EVALI)] [4,29,30]. The underlying mechanisms by which E-cigs contribute to lung disease remain incompletely understood, but it is likely that pathophysiologic effects on lung endothelial function are mechanistically involved. The endothelium is a critical site for inflammatory injury caused by a broad range of stimuli, including cell-based therapies [31] and a myriad of other processes. However, the injurious effects of E-cigs on lung ECs are only now beginning to be explored. Cortactin (CTTN) is an actin-binding protein that regulates cytoskeletal dynamics such as cell migration, adhesion, and apoptosis [14,17,31]. It plays a critical role in vascular integrity and has been studied in various diseases such as cancer and both acute and chronic lung disorders [22]. The potential mechanistic association between CTTN and E-cig-induced endothelial permeability is of particular interest given the essential barrier role of the lung endothelium [32,33]. In our previous work, we demonstrated that CTTN plays a critical role in regulating lung endothelial apoptosis and mitochondrial ROS. Specifically, silencing CTTN or blocking its SH3 domain significantly increased endothelial cell apoptosis, providing mechanistic insights into COPD pathogenesis [14,17,34].

### 3.2. Summary and Relevance of Current Observations

**Figure 6:**
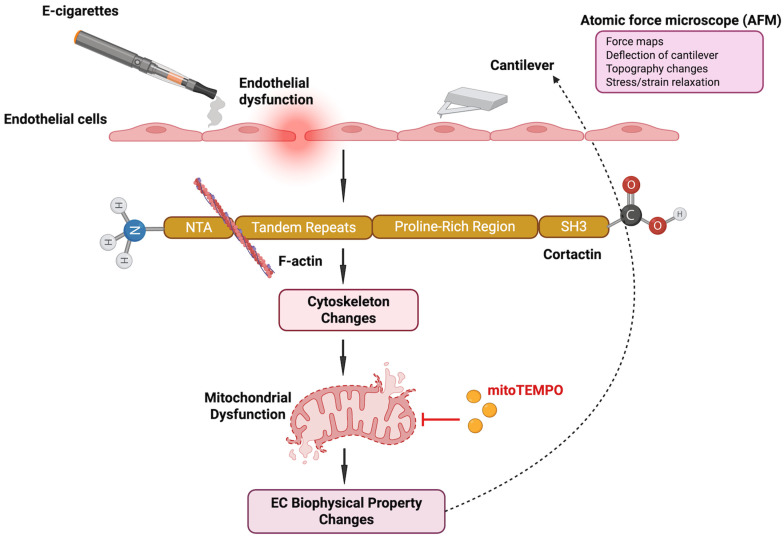
Schematic representation of the role of cortactin (CTTN) in e-cigarette-induced lung endothelial dysfunction. CTTN, an actin-binding protein, contributes to e-cigarette-mediated injury by promoting actin cytoskeletal rearrangement and mitochondrial dysfunction, leading to biomechanical changes. E-cigarette-induced barrier dysfunction is evaluated through the measurement of elastic properties using atomic force microscopy. The mitochondrial antioxidant MitoTEMPO mitigates these effects by reducing ROS production and preventing barrier dysfunction. This study highlights a previously uncharacterized mechanism of E-cigarette-induced lung endothelial barrier integrity and the role of CTTN in this mechanism.

Building on these findings, the current study integrates atomic force microscopy (AFM), trans-endothelial electrical resistance (TEER) permeability assays, and immunofluorescence imaging to examine how E-cigarette exposure alters cytoskeletal organization, cell–cell interactions, and the biomechanical properties of endothelial cells (Figure 6). The major findings of this study are that (i) E-cig induces loss of cell–cell interaction, causing gap formation in ECs; (ii) E-cig causes loss of endothelial barrier function and increases the elastic modulus (Y), indicative of increased cellular stiffness; (iii) EC “Y” is attenuated by pretreatment with MitoTEMPO (MT); (iv) EC “Y” is enhanced in CTTN-silenced ECs during E-cig challenge; (v) pretreatment with MT protects the endothelial barrier during E-cig challenge; and (vi) CTTN reduction by siRNA increases barrier disruption after E-cig challenge.

Mechanical properties regulate cell development and function, and the relationship between force generation and cell architecture is an emerging area for providing fundamental insights [35,36]. The current study focuses on exploring the role of CTTN structure/function (Figure 3 and Figure 5B) in modulating nanomechanical and rheological responses during E-cig-induced lung EC dysfunction [31,37]. External mechanical forces impact EC permeability through cytoskeleton rearrangement, junctional complex disassembly, and altered actomyosin contractility [18]. Here, we identify that E-cig exposure leads to loss of cell–cell interaction and increases in EC permeability (Figure 1 and Figure 2). To further investigate the role of CTTN and mitoROS in E-cig-induced lung EC dysfunction, AFM was utilized to characterize biomechanical properties in lung ECs after manipulation of CTTN expression/function (via siRNA) and ROS inhibitor (MitoTEMPO). The silicon nitride cantilever used for these AFM measurements assesses the rheological property of EC in a minimally invasive manner [38,39]. We employed AFM contact mode to generate force maps to calculate Young’s modulus and determine topographical changes in lung ECs upon E-cig or CS challenge. We hypothesized that reducing CTTN by siRNA, or inhibition of mitoROS generation with MitoTEMPO, alters E-cig responses in lung ECs in a manner that contributes to our understanding of their pathogenetic effects.

Our data indicate that E-cig exposure increases the elastic modulus in lung ECs (as estimated with the Hertzian model) compared to vehicle controls. Previous studies have shown that E-cig components such as glycerin and propylene glycol can induce cellular dehydration and osmotic stress, leading to increased stiffness and altered optical properties of endothelial and epithelial cells. These osmotic effects may contribute to the observed increase in elastic modulus in our study, suggesting that part of the stiffening response may arise from dehydration-related cytoplasmic changes. Future studies should evaluate these mechanisms in greater detail by referencing relevant work [40,41] and assessing hydration-related parameters alongside mechanical measurements.

Force–distance mapping assesses the deflection of the AFM cantilever as it approaches the sample, demonstrating that cantilever deflection is lower in E-cig compared to vehicle (Figure 5A–C). Reduction in CTTN by siRNA results in an increase in the elastic modulus, and this effect is accentuated further upon E-cig exposure (Figure 5B). In contrast, scavenging mitoROS with MitoTEMPO ameliorates this E-cig effect on elastic modulus levels, suggesting a functional role in E-cig-induced lung endothelial effects (Figure 5A). Elasticity is defined as a substance’s ability to resist deformation [42]. To complement our AFM data, we measured trans-endothelial resistance (TEER) using Electric Cell–Substrate Impedance Sensing (ECIS) to demonstrate that E-cig exposure reduces EC barrier function in a dose-dependent fashion when compared to vehicle (Figure 2). Reduction in *CTTN* expression by siRNA enhanced this pathophysiologic disruption of EC permeability by E-cig (Figure 3), while MitoTEMPO inhibition of mitoROS resulted in some EC barrier protection (Figure 4). The adhesive interactions between the AFM tip and the endothelial cell surface were below 50 pN, significantly lower than the applied loading force (~2 nN). Therefore, the Hertz model was chosen for our experimental analysis [43,44,45,46,47].

### 3.3. Limitations

There are several potential methodological limitations in our present study. First, while cantilever spring constants were determined using the thermal noise method, explicit calibration of the optical lever sensitivity (OLS) was not determined, which may affect absolute accuracy. Second, the Hertz model assumes perpendicular probe–sample contact, but small probe tilts inherent to the AFM holder design were not measured or corrected. Third, the use of optical microscopy to monitor the integrity of a 10 nm AFM probe is limited, as its resolution is insufficient to detect nanoscale tip blunting or contamination. To mitigate these limitations, tip integrity was assessed through the reproducibility of force–indentation curves and by replacing probes for each experiment. Nevertheless, direct nanoscale verification of tip geometry and more precise calibration procedures were not performed. These limitations can be addressed in future studies through explicit OLS calibration, tilt compensation, and direct tip characterization, for example, using scanning electron microscopy or reference substrates, to further improve the accuracy and reliability of AFM measurements. In addition, as noted above, it would be helpful for future work to assess hydration-related parameters alongside mechanical measurements.

### 3.4. Conclusions and Future Directions

In summary, these data indicate that E-cig exposure induces a series of pathophysiologic events in lung ECs, including increased elastic modulus, loss of intercellular interaction, decreased resistance, and increased EC permeability (Figure 6). Mechanistically, these responses are modulated in part by CTTN expression and mitoROS generation. These observations add to our previous work describing CS- and E-cig-induced apoptotic changes [14] and further support an important functional role for CTTN in mediating these responses. In addition, prior reports have demonstrated spatially specific alterations of the EC cytoskeleton in response to various agonists, with differential effects at the cell periphery, cytoplasm, and nucleus [12]. Further studies are therefore needed to measure the E-cig-induced deformations at different subcellular regions, such as the nucleus, cytoplasm, and periphery, to better understand the nanomechanical properties within the cell. Next steps may also include CTTN overexpression studies to determine if this approach is protective against E-cig-induced EC dysfunction. In vivo correlation is likewise needed and may include the use of genetically modified CTTN mice to study the effects of E-cigarette exposure on lung tissue and perform AFM measurements of lung tissue after E-cigarette exposure to assess changes in biomechanical properties. Future work may focus on assessing other EC biomechanical properties with AFM, such as height and other morphometric changes after E-cig exposure. These assessments can determine EC shrinking and loss of cell–cell interaction. Direct comparisons should be carried out between the effects of CS and E-cigs on endothelial cell biomechanics and permeability, and the differences and similarities of CS- and E-cig-induced changes in endothelial cells should be investigated. Finally, the potential signaling connection between CTTN structure/function and mitoROS needs further characterization and the effects of E-cig exposure on endothelial shrinking and loss of cell–cell interaction should be investigated.

## 4. Materials and Methods

### 4.1. Cell Culture

Human pulmonary artery endothelial cells (HPAECs) were purchased from Lonza (Walkersville, MD, USA) and cultured in Endothelial Cell Growth Medium-2 (EGM-2) (Lonza) supplemented with 10% fetal bovine serum (FBS) (Sigma, St Louis, MO, USA). Cells were maintained at 37 °C in a 5% CO_2_ incubator and used at passages 6–8 for all experiments. Cells were starved for 2 h in 2% FBS media before treatments.

### 4.2. E-Cigarette Preparation

“JUUL” e-cigarette was obtained commercially (JUUL, San Francisco, CA, USA). The 5.0% nicotine, unflavored pod was used for our experiments; it contains nicotine (5%), propylene glycol (35–45%), glycerin (~35–45%), and benzoic acid (0.2–2%). Cells were treated with 25–50 μg/mL of E-cig liquid for 24 h.

### 4.3. siRNA Transfection

HPAECs were transfected with scrambled RNA or *CTTN* siRNA (100 nM) using the DharmaFECT transfection reagent (Horizon Discovery, Lafayette, CO, USA), 48 h after transfection, EC were challenged with E-cig. Transfection efficiency was determined by Western blotting [14].

### 4.4. Immunofluorescence Microscopy

HPAECs were grown on an 8-well glass chamber slide to 80–90% confluence in EGM-2 medium. After indicated treatments, the cells were fixed with 3.7% paraformaldehyde for 10 min, followed by three washes with PBS. The cells were then permeabilized with 0.25% Triton X-100 for 5 min and rinsed with PBS for 5 min, followed by incubation in blocking buffer (1% BSA-PBS) for 1 h. The cells were then incubated with cortactin antibody for 1 h, washed with PBS, and then incubated with secondary antibody-Alexa Fluor 488 and Alexa 594-Phalloidin (F-actin staining) for 1 h. After washing at least four times, the coverslips were mounted with profound Gold DAPI (Invitrogen, Green Island, NY, USA). Images were taken using a Zeiss confocal microscope at 40× magnification (Dublin, CA, USA).

### 4.5. Reagents

Horseradish Peroxidase (HRP)-linked anti-mouse and anti-rabbit secondary antibodies, anti-β actin antibodies, and MitoTEMPO were purchased from Santa Cruz Biotechnology, Inc. (Santa Cruz, CA, USA). SiRNA (control and CTTN) and DharmaFECT1 transfection reagent were purchased from Dharmacon (Horizon Discovery, Lafayette, CO, USA). Anti-CTTN antibody was purchased from Sigma Aldrich, MO, USA.

### 4.6. Trans-Endothelial Monolayer Electrical Resistance (TEER) Measurements

Electrical cell impedance sensing (ECIS) (Applied Biophysics, Troy, NY, USA) was used to evaluate the integrity of the EC monolayer, as described in our previous work. Briefly, HPAECs were grown to confluence on gold microelectrodes, and the cells were subjected to a weak electric current for the continuous measurement of TEER. After 1 h of measurement (to establish a stable baseline resistance), the indicated stimuli were added to the wells, and measurement continued for 20 h. The data were analyzed using custom-designed Epool software by normalizing each resistance value to the starting resistance for that electrode, as we have previously described [37].

### 4.7. AFM Imaging

An Asylum MFP-3D-Bio atomic microscope (Oxford Instruments Asylum research, Santa Barbara, CA, USA) and PNP-TR-Au-1 triangular silicon nitride cantilevers (with tip height 3.5 µm and radius of curvature 10 nm) were purchased from Asylum Oxford Research Group. The nominal tip radius is from the manufacturer’s specifications. To ensure no contamination or tip blunting, we inspected the probe integrity before and after each experiment using optical microscopy. System alignment was performed to ensure probe orthogonality, which involved mechanical leveling using a bubble level placed on the detector assembly, as well as optical laser alignment on the cantilever via the built-in optical microscope. The laser beam was centered on the photodiode detector, and the deflection signals were balanced to zero when the cantilever was not in contact with the surface of the sample. The elastic modulus is quantified by the indentation of the cell’s surface by the cantilever tip to produce force–distance curves. Young’s modulus calculations were based on the bidomain polynomial models fitted to the experimental force curve using a standard least squares minimization algorithm. Imaging for cells in liquid was performed in contact mode. Cantilevers with spring constants of k = 0.08 N/m or 0.17 N/m were used for imaging and elasticity measurements. The spring constants were verified prior to each experiment using the thermal noise method. This method was used to confirm detector alignment and minimize noise. The mechanical properties of the cell cytoskeleton were acquired in tapping force mode. The tip velocity was set at 1.99 µm/s. The cell indentation part of the force curves was fitted to the Hertz model [48] and yielded Young’s elastic modulus as a measure of cell stiffness.

### 4.8. Statistical Significance and Data Analysis

All data are expressed as mean ± SEM from at least three independent experiments. Statistical analysis was performed using the GraphPad Prism 10 software. Student’s *t*-test or two-way ANOVA (Tukey’s or Dunnett’s post hoc tests) was used to compare two or more groups, respectively. Values of * *p* < 0.05 were considered statistically significant.

## Data Availability

Data is contained within the article or Supplementary Materials.

## Supplementary Materials

The following supporting information can be downloaded at: https://www.mdpi.com/article/10.3390/ijms262412104/s1.

## Abbreviations

AFM	Atomic force microscopy
CDC	Centers for Disease Control and Prevention
COPD	Chronic obstructive pulmonary disorder
CS	Cigarette smoke
CTTN	Cortactin
ECs	Endothelial cells
ECIS	Electric cell–substrate impedance sensing
EVALI	E-cig-induced acute lung injury
E-cigs	E-cigarettes
FDA	Food and Drug Administration
HRP	Horseradish peroxidase
HPAECs	Human pulmonary artery endothelial cells
mitoROS	Mitochondrial reactive oxygen species
MT	MitoTEMPO
OLS	Optical lever sensitivity
TEER	Trans-endothelial electrical resistance
Y	Young’s modulus
