# Supplementary material for: Nanomechanical Characterization of E-Cigarette-Induced Lung Endothelial Dysfunction: Roles of Cortactin and Mitochondrial Reactive Oxygen Species
Source: Int J Mol Sci. 2025 Dec 16;26(24):12104. doi: 10.3390/ijms262412104 (PMC12733622; doi:10.3390/ijms262412104)
Supplement: Supplementary file 1 [file ijms-26-12104-s001.zip › ijms-3936418-supplementary.pdf]

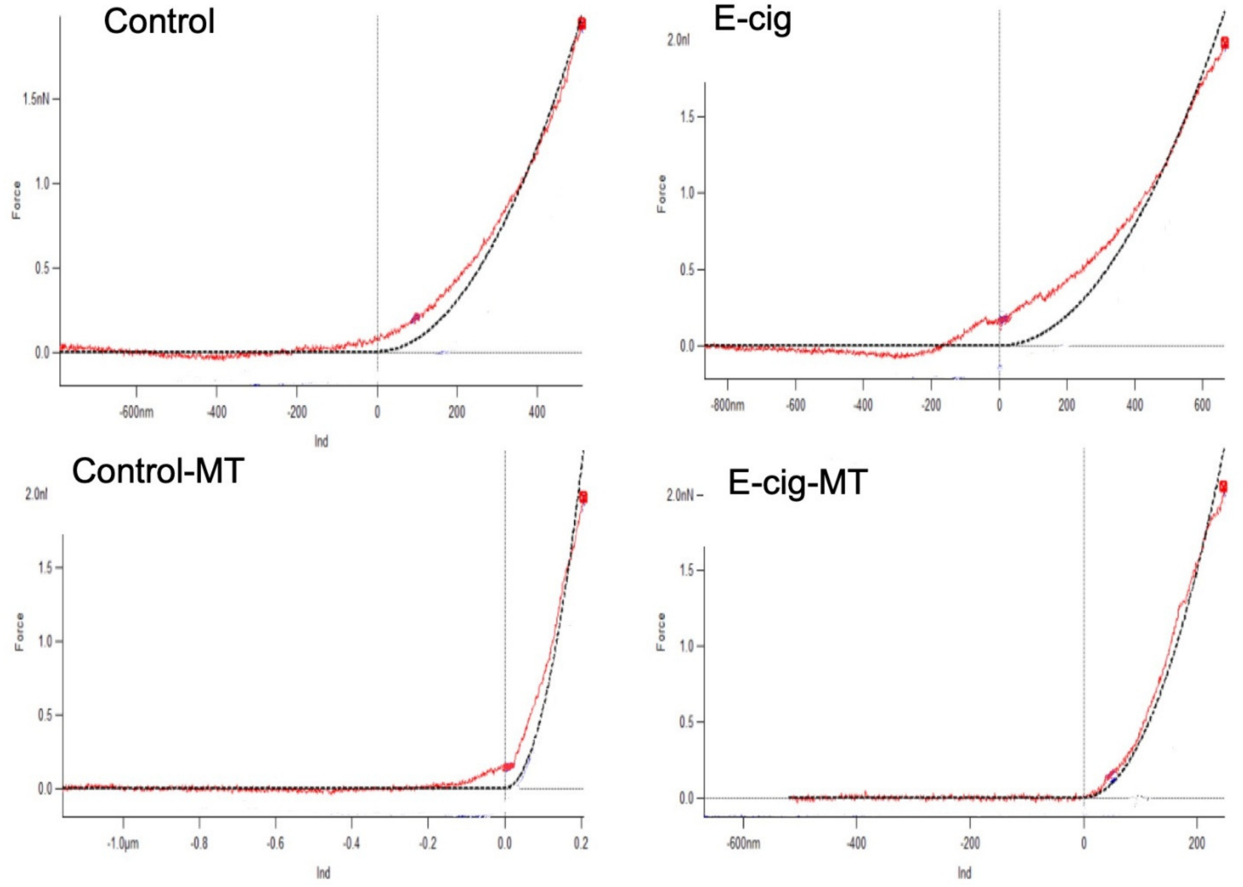

Supplementary Figure 1: **Force vs Deflection Curves-**Representative force–deflection curves for each condition used to measure the elastic modulus in HPAECs are shown. The four conditions include: Control, E-cigarette exposure, MitoTEMPO, and MitoTEMPO followed by E-cigarette exposure (20 μM, 3 h)

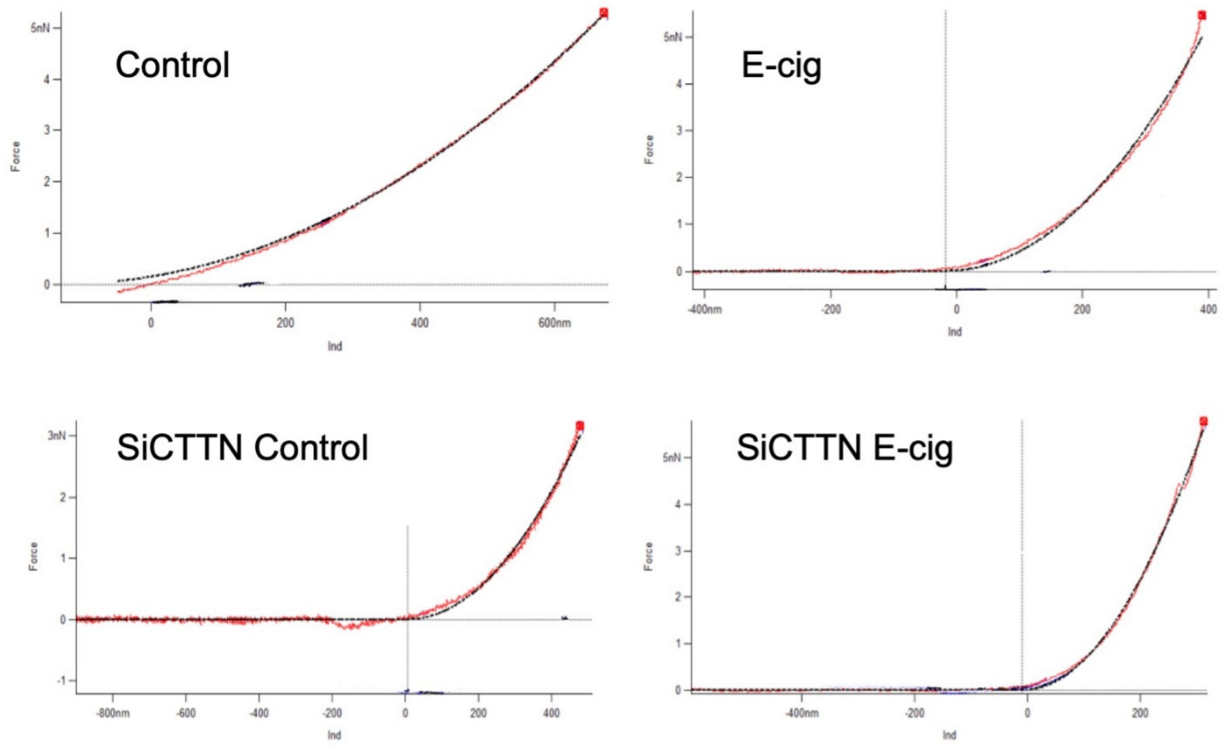

Supplementary Figure 2: **Force vs Deflection Curves-**Representative force–deflection curves for each condition used to measure the elastic modulus in HPAECs are shown. The four conditions are: Control, E-cigarette exposure, SiCTTN, and SiCTTN followed by E-cigarette exposure.
